# ESR white paper on teleradiology: an update from the teleradiology subgroup

**DOI:** 10.1007/s13244-013-0307-z

**Published:** 2014-01-18

**Authors:** European Society of Radiology (ESR)

**Affiliations:** Neutorgasse 9/2a, 1010 Vienna, Austria

**Keywords:** Teleradiology, Teleradiologist, Teleradiology company, Quality of care, Regulatory issues, Patient primacy, Technology, European community, Outsourcing

## Abstract

**Background:**

Teleradiology services are increasingly integrated into the workflow of radiological departments in EU-member states.

**Methods:**

The current technological possibilities and European political agenda are both opening the way for cross-border telemedicine services including teleradiology.

**Results:**

This is bringing new opportunities for both users and providers of teleradiology services, which has led to the idea of producing an updated version of earlier ESR statements and communications on teleradiology. For this purpose the e-Health and Informatics subcommittee established a Teleradiology subgroup.

**Conclusion:**

This white paper proposes comprehensive best-practice guidelines for teleradiology usage, focussing on services within the European Union, as prepared by the members of the ESR teleradiology subgroup.

***Main Messages*:**

• *Teleradiology describes the provision of radiology services remote from the site where the images are obtained.*

• *Teleradiology should form part of and be integrated with the wide spectrum of radiology services, and not a separate tradable commodity*

• *The quality of radiological reports and services delivered by teleradiology should not be less than those of local radiologists*

• *International quality standards for teleradiology need to be established*

• *Patients need to be fully informed when teleradiology is used.*

## Introduction

This White Paper is intended as an update of the document “Teleradiology in the European Union-White Paper”, issued by the European Society of Radiology (ESR) in 2006 [1]. The process of digitisation is progressing quickly within health care and the European political agenda is firmly embracing this digital revolution [2]. Telemedicine and e-Health services are being promoted on a pan-European level, causing major changes in European legislation. These evolutions impelled the e-Health and Informatics subcommittee of the ESR to create a Teleradiology subgroup tasked with writing a teleradiology White Paper. A table of contents was first developed to help guide this effort; consecutively leading authors were assigned for each chapter. Teleradiology subgroup members were able to share and discuss their individual contributions using Google Drive (http://drive.google.com). Editing of the individual contributions of the Teleradiology subgroup members finally resulted into this more concise version.

Teleradiology can be of great value, specifically in areas deprived of highly specialised medical care and diagnostic services, but it also has a number of inherent limitations. Its limitations and role need to be set into the context of radiologists’ wider responsibilities. These include the need to oversee the delivery of high-quality and efficient local imaging, the justification, development and optimisation of imaging protocols to protect patients from unnecessary investigations or inappropriate and unjustified radiation exposure. Direct discussion of cases individually with clinical colleagues and contribution to multidisciplinary meetings are important aspects of radiologists’ work, which have increased significantly in recent years. These meetings are often the most effective way to develop an understanding of the relevance of imaging findings to patient management, leading to continuous improvement in the quality and clinical usefulness of reports. Face-to-face teaching of trainees and junior doctors is also important, as is the vital provision of interventional radiology, particularly for emergency cases. In the commissioning of teleradiology services, the need to maintain a ‘critical mass’ of radiologists who can provide these services locally must be addressed and should not be compromised.

## Definitions

*Teleradiology* is the exchange of radiological images and patient-related data between geographically different locations for purposes of primary interpretation, expert consultation and/or clinical review by digital transmission. This process also involves the digital sharing of patient-identifiable information, within and among different organisations and, in some situations, across national boundaries. Typically the interpreting *teleradiologist* works from a location other than that where the patient is being examined. A *teleradiologist* can be defined as the physician providing the reading services, and a *teleradiology company* is the entity contracting one or more teleradiologists and engaging in the management of workflow and image distribution [3]. The site where the images have been acquired is referred to as the *transmitting site*, and the site at which a reading is provided is the *receiving site*.

Teleradiology services can be divided into *intra*-*mural teleradiology* (intra-organisational, in-sourced) and *extra*-*mural teleradiology* (extra-organisational, out-sourced). The distinction between both types of services is hereby based upon the organisation-patient and doctor-organisational relationship. If the teleradiologist is employed by or working for the organisation that has a direct relationship with the patient, services delivered are considered as *intra*-*mural*, even if readings are performed from a different location or country. All medical and imaging activities fall under the same governance and leadership, and the teleradiologists are subject to the same policies and procedures as radiologists working on site. In this model the teleradiologist should have full access to the EPR (Electronic Patient Record) as if he/she were working in the radiology department of that same institution. *Extra*-*mural teleradiology* is taking place when the interpreting teleradiologist is working for a teleradiology company, usually not affiliated to the hospital or institution that is providing care for the patient. Access to the EPR might not automatically be available in this model. For imaging studies being transmitted cross-border (EU, world), the terminology *international teleradiology or cross*-*border teleradiology* seems appropriate. Distinction should be made between international teleradiology *within* vs. *outside EU*-*borders* since for the latter no specific regulatory framework is available.

On the service level a distinction can be made between different types of teleradiology depending on the goals of the services provided. We propose the following categorisation:

- Preliminary reads
- Radiological image interpretation usually performed in on-call emergency situations where there is no local radiologist available. The on-site radiologist composes the final authenticated report, usually the next day during daytime office hours.
- Primary reads
- Image interpretation is performed with the purpose of making a radiological diagnosis, in both emergency and non-emergency situations. A final radiological report is delivered by the teleradiologist and not by the radiologist on-site.
- Second opinion and expert opinion
- Image interpretation should be requested by the local radiologist or clinician (peer-to-peer consultation) when there is doubt about a specific diagnosis. This usually occurs when specific in-house knowledge about a radiological subspecialty is insufficient or unavailable. When a radiologist with a specific expertise is consulted this should be called an *expert opinion*. When a clinician asks a (local) radiologist for a full report of a particular examination performed elsewhere, this should be called a *second opinion*.

## Prior ESR statements and comments on teleradiology

Several ESR documents have already addressed the topic of teleradiology. A first position paper was released in 2004 by the European Association of Radiology (EAR) in conjunction with the Radiological Section of the Union of European Medical Specialists (UEMS), re-issued as the ESR position paper on teleradiology in 2006 [4]. In this paper potentially problematic teleradiology-related issues were highlighted in the field of communication, access to priors, quality control and legal ambiguities. Guidelines were provided for teleradiology usage, stressing that it should only be used for the benefit of the patient and not diminish the quality of services.

In November 2006 the ESR-UEMS White Paper “Teleradiology in the European Union” was published [1]. The principal reasons for the publication of this document were the recent teleradiology developments in Europe, the advertising of teleradiology services from the Far East and a report issued by the American College of Radiologists (ACR) Task Force on Teleradiology Practice, in which legitimate questions were posed concerning the use of cross-border teleradiology with regard to the quality of patient care [5]. Some of the original key ESR/UEMS recommendations were re-emphasised stating that:

- Clinical teleradiology is an integrated medical service
- The “source radiologist” (on site radiologist) has to be involved in the decision-making process of “outsourcing”
- A quality of standard regarding teleradiology equipment is required
- A quality control system, such as clinical auditing, is needed
- A more uniform EU legislation is needed safeguarding the patients’ rights

In 2009 the ESR published its Response to the EC communication on telemedicine COM(2008)689, in which the ESR recognises the usage of teleradiology as a reality that is likely to expand, but for which legal and medical concerns still exist [6–8]. According to the ESR, politicians should make a distinction between telemonitoring and teleradiology, the first being a simple health care service and the latter being a well-established expansion of an already existing medical practice, i.e. radiology, requiring a radiologist. Furthermore the ESR stresses the importance of developing an EU-wide legal framework, of fully integrating teleradiology services in clinical practise and of making broadband ICT infrastructures available for safe distribution of patient data.

In May 2011 an ESR response to public consultation on the e-Health Action Plan (eHAP) 2012-2020 was released, in which the ESR repeated that “*Teleradiology should be explicitly defined as a medical act in order to ensure quality of care and patient safety*…” and that “…*the same level of guarantee*, *in terms of quality and safety*, *must be applied to these services as compared to standard medical acts*” [7].

In summary, the ESR wants a future EU legislation to provide the following:

- Definition of teleradiology as a medical act in its own right
- Establishment of EU-wide accreditation criteria for teleradiology providers
- Emphasis on the importance of delivery of high-quality health care
- Application of international quality standards including monitoring of service providers
- Regulation of teleradiology as a responsibility of the member state where the patient undergoes the imaging procedure
- Full information of patients and informed consent about usage of teleradiology

European legislation is complex; for this reason a comprehensive analysis of the different legal aspects was released in December 2012 in the Telemedicine Commission Staff Working Paper on the applicability of the existing EU legal framework to telemedicine services [9]. Based upon this publication, the ESR has recently published the “ESR statement on the legal aspects of Telemedicine” [10].

## Teleradiology in Europe: current status

Recent developments in telemedicine and e-Health services are causing a major shift in the traditional methods of providing medical care. The growing cost of health care has put e-Health high on the political agenda: the “Europe 2020 vision” is opening the way for e-Health services since they are believed to have the potential to both reduce costs and improve the quality of health care. Due to the predominant digital character of medical imaging, radiology is on the forefront in this new e-Health scene. For radiologists, telemedicine is equivalent to teleradiology.

It has become commonplace to exchange large data sets with radiological images and related patient information between display stations both within and outside imaging facilities and hospitals using the Internet and other high-speed data links. In several European countries, networks have been established between hospitals within or between regions to facilitate this type of information exchange. Several commercial teleradiology service providers are active in the European market, some of them on a cross-border basis. This evolution is gradually changing the European radiology scene, creating more competition on an international scale [11]. Compared to the US, the usage of commercial teleradiology services in the European Union has remained relatively limited. Some member states however are being confronted with a shortage of radiologists and several market analysts are expecting a growing demand for non-invasive diagnostic imaging with an increasing demand for highly specialised radiological expertise. Therefore it is expected that usage of teleradiology will increase in the coming years. One of the causes for the relatively slow deployment of commercial teleradiology in Europe is most likely the fear that reliance on teleradiology companies might drive down reimbursements for radiology through price competition. Proof of such a fear is the publication in 2011 of a Teleradiology Charter by the French radiologists, as a reaction to commercial telemedicine companies undercutting the national tariffs [12]. Another reason might be the fear that further commoditisation will automatically result in a reduced quality of health care and thus threaten radiology as a medical specialty. It is therefore essential to define standards of good practice in order to maintain high-quality diagnostic imaging services [11]. Implementation of such standards will help decision makers to find a good balance between quality and pricing of teleradiology services [3, 11].

## EU legal framework

Besides the ESR Standards, other standards have been established (IRQN, ACR), none of which are supported by a legal requirement for implementation [13].

Following the so-called “subsidiarity principle”, teleradiology is the responsibility of national governments when undertaken within the borders of an individual country and is subject to the legal constraints within that country [14]. Most Member States (MSs) do not have legal instruments dealing specifically with teleradiology and only a few have regulations or guidelines. In Germany a legal standard for teleradiology was created (Röntgenverordnung, RöV), and the German Radiology Standards Committee NAR published a DIN-standard for quality assurance in teleradiology. European legal involvement is only relevant when cross border teleradiology is utilised, which increases the complexity of the delivery of the service. In fact, the EU citizens' Report 2010 revealed that fragmented legal rules on essential aspects of health care across MSs, hamper patients’ rights to receive health care in other MSs and caused concern for health care professionals [15]. In May 2013 the “ESR statement on the legal aspects of Telemedicine” was released [10]. We will assess the most essential parts of this statement; the full text can be downloaded from the myESR website.

### Licensing and registration

The European directive requires that teleradiology is provided in accordance with the legislation of the teleradiologist’s Member State (MS) of establishment and not with the legislation of the MS where the citizen (patient) underwent the imaging. As a result, the teleradiologist has responsibility to the professional registration authorities of the MS where he/she performs the interpretation and is not required to register with the patient’s MS authorities.

### Teleradiology as a medical act

Telemedicine is not a new medical act, and it is not intended to replace traditional methods of care delivery, such as face-to-face consultations. Rather, it represents an innovative way of providing health care services, which can complement and potentially increase the quality and efficiency of traditional health care delivery. All MSs that do not make teleradiology a medical act should be pressured to do so.

### Patients’ Rights

Patients have the possibility to receive treatment (i.e. radiological interpretation and diagnosis in teleradiology) in another MS and can be reimbursed under certain conditions. They are entitled to receive a report, which the teleradiology service should send directly to both the patient and the referring doctor. This report should include the details of the investigation and the name of the individual who interpreted and reported on the examination. Upon request relevant information on the standards and guidelines on quality and safety in the MS of the teleradiology company (MS of treatment) should be made available from the national contact point of that MS. The MS of treatment must ensure that teleradiology providers provide relevant information, including the availability, quality and safety of the service that is used, and that they also provide the interpretation and clinical advice in teleradiology (health care) as well as information on their authorisation or registration status. It is clear that if a patient suffers harm he/she may take legal action in his or her own MS of domicile. Systems of professional liability insurance or a guarantee of similar arrangement need to be in place.

### Informed consent

It is the view of the ESR that informed consent can only be obtained if the patient is informed at the site of imaging that their images may be interpreted through a teleradiology service. In addition, the patient should be informed of all the above provisions, including the reporting radiologist’s qualifications, prior to their agreement to accept the service.

### Liability

The legislation of the Member State in which the teleradiological provider is established should apply as a rule to the provision of cross-border teleradiology services to patients. Where a contract exists, either between professionals in one country and the teleradiologists in another MS or between imaging centres or hospitals and the teleradiology service in another MS, the general rule is the applicable law to the contract will be the one expressly chosen by the parties. In the absence of choice of applicable law in the contract, the contract of provision of services shall be governed by the law of the MS where the service provider has his legal residence.

### European diploma

The ESR recommends teleradiologists to take the *European Diploma in Radiology* (EDiR, http://myebr.org) organised by the European Board of Radiology. It provides an objective test of knowledge and skills for radiologists at the end of training as laid out in the European Training Curriculum for Radiology [16]. In the context of increased professional mobility and increased use of teleradiology, the EDiR will ensure harmonised knowledge, skills and competences and be a valuable proof of knowledge for the teleradiologist towards authorities, contractors and patients, in particular given the current legal uncertainties in Europe regarding the registration and accreditation requirements.

## Technology-related issues

Once digital radiology images are acquired they are stored in local servers within an organisational or enterprise PACS (Picture Archiving and Communication System). More recently, there has been use of off-site archiving of images, often referred to as “cloud”-based storage and Vendor Neutral Archiving (VNA). Secure storage of images and data remains the responsibility of the organisation and is subject to the legislation of each EU member state. Technical implementations for teleradiology applications must provide robust solutions reliably protecting patient data and offering sufficient network bandwidth to work efficiently and meet contractual agreements. For teleradiology purposes there are additional requirements regarding workflow support and availability of relevant medical information. Ideally the teleradiologist should have access to

- Radiological images without loss in quality during transmission or display
- Clinical referral information: referring letter or request card
- All relevant prior images
- Access to clinical information such as blood results, pathology reports and clinical correspondence

Several technical solutions are available, all of which have their advantages and disadvantages. An overview of the most common teleradiology settings is given below, including some recommendations regarding the teleradiology working environment.

### Common methods of secure transmission

- Virtual private network (VPN):

Teleradiologists may access the RIS-PACS System of the transmitting site via a VPN, which ensures secure transmission of data outside the institution-secured network and protection of patient privacy from unpermitted external access. This type of teleradiology is often used for on-call situations, allowing radiologists to interpret the images from home. The main advantage is that there is no risk of patient data duplication in multiple IT Systems within the network, and the radiologist has access to relevant patient-related information. The organisation must comply with legal requirements of patient confidentiality, related to commercial teleradiologists having access to entire enterprise PACS-RIS databases, if they are not employed by the organisation.

- Data push technology:

Several techniques exist for pushing data (images and relevant information) between the host RIS-PACS systems at the transmitting site and the receiving teleradiology system. When using *Direct DICOM Push* the images are directly pushed from the PACS of the transmitting site to the receiving site’s PACS. Referral letter or electronic order & scheduling information may be pushed from the transmitting site’s RIS to the receiving site’s RIS using HL7 order messaging (ORM). Once a report has been generated it can be pushed to the transmitter’s RIS and PACS via HL7-ORU messaging. Direct DICOM push is dependent on a PACS system to be present at both the transmitting and receiving site, with possible inherently related problems such as data redundancy and limited patient data security on the receiving PACS’ site.

DICOM images can also be sent or pushed via other secure transport systems. *DICOM e*-*mail* and *secure web services* allow for DICOM images to be transported without the need for a PACS system being present. *DICOM e*-*mail transmission* is currently the standard system for teleradiology in Germany, which is accepted by the regulatory authorities [17].

- Other transmission techniques:

Setting up a DICOM push communication including HL7 connections can be very time consuming and cumbersome, mainly because of differences in the interpretation of the standard by vendors. Other transfer protocols such as XDS and XDR use minimal metadata and support different document formats for point-to-point transport of images and reports. It is hoped that with adoption these IHE-supported protocols will help easier exchange of documents and images between health care institutions.

### Teleradiology work environment and ergonomics

The teleradiology service provider is responsible for providing an appropriate working environment, including state-of-the-art computers, monitors and viewing software certified for diagnostic purposes. An excellent online reference document for practicing radiologists about technical standards is the ACR-IT Manual [18].

The quality of the images should be preserved by all means. There should be no loss of data in the transfer of images from the transmitting site to the receiving site. Monitor and graphics cards used by radiologists on their reporting workstations must have regular quality assessment and must be fit for the purpose of diagnostic display. In order to preserve maximum image quality specific attention should be given to room lighting. Adequate ambient light preventing glare and potential interference with the optimal image quality of diagnostic monitors should be used, preferably with an intensity approximating the monitor brightness. Ambient lighting conditions become even more important with 5-megapixel monitors, which are accepted as a standard for mammography readings and in general have a lower brightness [19]. Teleradiologists should ensure that they have control over the ambient light in the reporting room [20, 21]. LCD displays in a three-monitor configuration are most acceptable. The brightness of the monitor is as important as image resolution, and DICOM grey-scale calibration should be used to decrease the need to perform windowing/levelling [22].

One of the principal goals of ergonomics in teleradiology is the prevention of repetitive stress injuries, visual and even mental disturbances [3, 19–23]. It is therefore essential that adequate measures be taken to maximise comfort and safety for the teleradiologist [21]. Emphasis should be placed on training users to make the best use of equipment [22]. In teleradiology, specific attention should be paid to specific privacy and security protocols of the “digital” working environment [3, 23]. Teleradiologists should be provided with the time, facilities and environment so that reporting can be carried out under ideal conditions in the interests of both accuracy and efficiency. Frequent breaks are required to prevent fatigue, particularly when reporting complex examinations [23, 24].

## Communication in teleradiology

### General principles

Effective two-way communication between the referring doctor and the interpreting teleradiologist is as essential in teleradiology as it is in an in-house setting. Reporting must be considered a dynamic process. If the referring clinician is unsure about the report or has doubts about the findings, this should be discussed with the teleradiologist who should have tools available to add an addendum based upon information from the clinical discussion. Moreover, all radiological opinions given verbally must be documented. Efficient communication with radiographers is necessary to guarantee the best applicable imaging strategy. Protocols regarding communication of urgent findings need to be established. Ideally, reports containing urgent information should also contain return receipts of asynchronous communications. Communication lines should be robust, easily accessible and the subject of a mutual contractual agreement.

### Clinical information and priors

The availability of adequate clinical information and a clear clinical question in the request form is essential in any radiological setting. The teleradiologist must be able to consult the patient’s previous imaging history and associated reports before providing a report [24]. In addition, the teleradiologist must have access to the patient’s demographics (gender, age), name and contact information of the referring physician and an emergency number to be contacted out of office hours.

When confronted with an examination request, the teleradiologist should first evaluate whether the quality of the information provided (images, priors if required, and clinical information) is sufficient to adequately answer the clinical question. If the teleradiologist feels the quality of images and information is insufficient and if it is impossible to obtain additional information, this should be stated in the report [24]. Recommendations or advice on further management, including new investigations or referral to a different specialty, must be appropriate for the clinical setup in which the patient is being treated; the teleradiologist should therefore be familiar with local clinical practice [3].

### Language and structure of the report

In a cross-border setting the report must fulfil the local legal language requirements of the country of the patient. Experience has shown that even reports in English by non-native English speakers can be confusing because of non-standard wording. Ideally, the obligation only to hire the services of teleradiologists entitled to practice in the country of the referring clinician (or within the European Union, where applicable) should be completed by generic training and certification of the teleradiologist’s ability to deliver unequivocal reports in the language of the patient.

In the future, structured reporting may help to overcome the language problem in international teleradiology. Efforts made by scientific societies to develop structured reporting systems, e.g. the RSNA/ECR Structured Reporting Initiative, should therefore be encouraged.

## Contracting and financing

In this paragraph recommendations that can be used for contracting teleradiology services within the European Union are given. However, the organisation and delivery of health care are the responsibility of each individual EU Member State; therefore it is possible that national legislations require additional or specific demands regarding the usage of teleradiology services, including reimbursement and terms and conditions of contracts [25–27]. As stated previously, teleradiology is part of the medical specialty radiology and should not be considered as separate service, technology or specialty. Therefore the teleradiology provision contract should adhere to the same principles as for the provision of in-house radiology services. It should be transparent for the patient who is responsible for each particular step of the examination and diagnostic pathway [28].

In the vast majority of European national health insurance systems there is no specific reimbursement fee or tariff available for teleradiology services. Usually the cost price for teleradiology is based upon a mutual agreement between the transmitting site and the teleradiology service provider (receiving site, teleradiology company), and in most cases it is financed as part of the integral radiology budget. Several business models exist in teleradiology, varying from a simple bilateral connection to a complex many-to-many type of connection using a teleradiology brokering service. In the contract the purpose of the service, responsibilities, liability and other relevant issues should be addressed [3, 27]. Teleradiology services should not be regarded as a tradable commodity but as a strategic part of the entire imaging process. The local radiologists should always be involved in the decision-making process of outsourcing with teleradiology. The financial and/or reimbursement issues should be discussed transparently with all parties involved, including radiologists, and the pricing should never be the principal basis upon which a decision to outsource is made. Therefore the contract with a teleradiology provider should also include clear and understandable incentives for the local radiologists and local medical staff [29].

From the clinical perspective the essential elements that should be addressed in the contract are the availability of the referral letter, access to prior images and EPR, imaging quality assessment and communication procedures. In the service level agreement (SLA), which is part of a service contract, the details of the service should be clearly defined. This includes but is not limited to topics such as the type of the report (preliminary report or emergency reading, second opinion, final report, usage of structured reporting), hours and times of teleradiology coverage, turnaround times (TATs), volumes and types or complexity of examination.

From the technical point of view the contract should address network security issues, for which local legislation concerning transmission of patient-sensitive data needs to be taken into account. Confidentiality and privacy issues are to be covered sufficiently. Licensing and accreditation of the teleradiologist need to be an integral part of the contract.

In summary it needs to be stressed that teleradiology outsourcing should only be used in case there is a clear need and benefit for the patient and that local radiology professionals have to participate actively in the decision process [3, 27, 29]. Equally the health care professionals have to make sure that they are working within the proper regulatory framework and meet all legal and regulatory requirements, independent from the level or type of teleradiology services.

## ESR initiatives in teleradiology training

Given the rapid advances in the field of digitisation and related changes to radiology practice as well as potential legal implications, teleradiology and telemedicine should be considered as an integral part of residency training. The European Training Curriculum for Radiology (former European Training Charter for Clinical Radiology) of the ESR is designed to provide a valuable template for training radiologists and to enhance the quality of care for patients throughout Europe [16]. In response to the increased use and fast developments in the fields of telemedicine and e-Health, the revised curriculum includes knowledge and skills in teleradiology as part of the Level I Training Programme (years 1-3). Teleradiology is covered both within the topic of Imaging Informatics as well as in the area of Communication and Management in order to convey the principles of teleradiology and its potential role and legal implications. In addition the ESR envisages providing a training platform for teleradiology, e.g. by developing courses and workshops on good practices in teleradiology as well as European regulatory initiatives at the ESR Learning Centre in Barcelona (http://www.esrlearningcentre.com). The training platform will allow teleradiologists to keep abreast of IT developments, learn about European legislative and project initiatives as well as to share experiences.
